# *GF14f* gene is negatively associated with yield and grain chalkiness under rice ratooning

**DOI:** 10.3389/fpls.2023.1112146

**Published:** 2023-02-10

**Authors:** Feifan Lin, Sheng Lin, Zhixing Zhang, Wenxiong Lin, Christopher Rensing, Daoxin Xie

**Affiliations:** ^1^ Tsinghua-Peking Joint Center for Life Sciences, and MOE Key Laboratory of Bioinformatics, School of Life Sciences, Tsinghua University, Beijing, China; ^2^ Fujian Provincial Key Laboratory of Agroecological Processing and Safety Monitoring, School of Life Sciences, Fujian Agriculture and Forestry University, Fuzhou, China; ^3^ Key Laboratory of Crop Physiology and Molecular Ecology, Fujian Agricultural and Forestry University, Fuzhou, China; ^4^ Institute of Environmental Microbiology, College of Resources and Environment, Fujian Agricultural and Forestry University, Fuzhou, China

**Keywords:** ratooning season rice, GF14f gene, carbon reserve remobilization, starch composition, oxidative and environmental resistance

## Abstract

**Background:**

Ratoon rice cropping has been shown to provide new insights into overcoming the current challenges of rice production in southern China. However, the potential mechanisms impacting yield and grain quality under rice ratooning remain unclear.

**Methods:**

In this study, changes in yield performance and distinct improvements in grain chalkiness in ratoon rice were thoroughly investigated, using physiological, molecular and transcriptomic analysis.

**Results:**

Rice ratooning induced an extensive carbon reserve remobilization in combination with an impact on grain filling, starch biosynthesis, and ultimately, an optimization in starch composition and structure in the endosperm. Furthermore, these variations were shown to be associated with a protein-coding gene: GF14f (encoding GF14f isoform of 14-3-3 proteins) and such gene negatively impacts oxidative and environmental resistance in ratoon rice.

**Conclusion:**

Our findings suggested that this genetic regulation by GF14f gene was the main cause leading to changes in rice yield and grain chalkiness improvement of ratoon rice, irrespective of seasonal or environmental effects. A further significance was to see how yield performance and grain quality of ratoon rice were able to be achieved at higher levels via suppression of GF14f.

## Introduction

China is the largest global producer and consumer of rice (FAO, 2020). This is especially true regarding Southern China, which makes the greatest contribution to both China's rice industry and global rice production (Lin et al., 2022). However, Southern China has now been forced to significantly change its practices because light and temperature resources in this region are enough for one seasonal crop but insufficient for two seasons (Lin et al., 2015). To counteract this challenge, ratoon rice cropping was introduced for greater rice production and has become increasingly popular in Southern China (Xu et al., 2021). Rice ratooning refers to a regenerative phenomenon of rice where new tissues can develop from dormant buds of stem nodes on residual stubbles after harvesting the main crop (Plucknett et al., 1970), and is the hallmark of more green and economic rice production (Shen et al., 2021; Yu et al., 2021; Xu et al., 2022). Modern forms of ratoon rice cropping originate from Texas and southern Louisiana, USA, having had a long history there beginning in approximately 1927 and then expanding to Asian countries in 1970 (Wang et al., 2021). In the past decade in China, ratoon rice cropping has been widely disseminated due to the Chinese government actively promoting the practice and introducing several policies, such as the Project of High-Yield Cultivation Techniques for Ratoon Rice (2009-2010), the Project of Comprehensive Cultivation Techniques for Ratoon Rice (2015-2016), and the Project of High-Yield and Highly Efficient Cultivation Techniques for the Mechanized Harvesting of Ratoon Rice (2017-2018). Through the use of this system, an average increase in yield was achieved, with the yield advantages of ratoon rice cropping jumping from 26.1% to 71.43%, as compared to the traditional cropping system (Firouzi et al., 2018; Yuan et al., 2019). Not only that, ratoon rice was able to meet the fast-growing market demand for grain quality in the global rice industry (Fitzgerald et al., 2009). It has been documented that ratoon rice cropping significantly contributes to a distinct improvement in rice quality. For example, the physicochemical properties and textural characteristics of cooked ratoon rice were highly improved when compared to the main crop (Deng et al., 2021). Zhou (2006) and Wu (2005) claimed a significant decrease in chalky grain percentage in ratoon rice as compared to the main crop. Recently, it has been documented that ratoon rice significantly reduced grain chalkiness as compared to late-season rice, indicating that such an improvement in the chalkiness of ratoon rice might be irrespective of the seasonal effect (Huang et al., 2020). However, the potential molecular mechanism remains unclear. Despite that, there is compelling evidence to claim the significance of ratoon rice cropping for higher yield and better quality rice.

Grain filling is the final stage of growth for cereals with a duration that continues into maturity. Grain filling will initiate source-to-sink transport for delivering the carbon and nitrogen substances into caryopsis (Cruz-Aguado et al., 1999) and, as a result, determine the yield and quality of cereals (Zhang et al., 2021; Wang et al., 2008; Shimoyanagi et al., 2021). However, both source-to-sink transport and grain-filling properties are distinctly different in the comparisons between ratoon rice and traditionally-cultivated rice. Rice ratooning can regenerate new spikes from auxiiary buds on the residual stubbles and break into the grain-filling stage instead of undergoing a long-day vegetative growth period (Lin et al., 2015). This means that there is an inherent pre-start and speed-up for senescence occurring in ratoon rice. It was previously pointed out that a whole senescence sequence of cereal is required to remobilize and transfer assimilates pre-stored in vegetative tissues to grains (Yang and Zhang, 2006). It has been widely accepted that the role of plant hormones, especially regarding abscisic acid (ABA), determines the intimate relationship between senescence and carbon reserve remobilization (Yang et al., 2002; Yang et al., 2006; Wu et al., 2008). For example, ABA promotes the transference of carbohydrates from stem to grain by regulating the relative gene expression in carbon reserve remobilization (Wang and Zhang, 2020; Wang et al., 2020). Meanwhile, the sucrose and abscisic acid interaction affected the activities of key enzymes in sucrose-to-starch conversion (Tang et al., 2009; Wang et al., 2015), thereby regulating the grain-filling process. Abscisic acid, a kind of dominant hormone in rice senescence (Mao et al., 2017; Sakuraba et al., 2020), was documented to be enriched in the source tissues of ratoon rice at post-anthesis while displaying low levels in their counterparts of the single-cropping late rice (Zhou, 2020). These results indicated a potential ABA-dominated senescence with additional effects of carbon reserve remobilization and grain filling process underlying rice ratooning. Except for ABA-associated senescence, environmental stimuli are also a major contributing factor inducing the senescence process in rice (Yang et al., 2001a; Yang et al., 2001b; Yang et al., 2002; Kim et al., 2011; Prathap et al., 2019; Ren et al., 2021). Indeed, controlled environmental stress helps in initiating the remobilization of carbon reserves and thereby greatly contributes to an increase in rice yield and grain quality (Yang et al., 2003; Yang et al., 2004). However, ratoon rice might also exhibit poor grain filling as traditionally-cultivated rice, particularly in the inferior spikelets (Yang et al., 2000; Ishimaru et al., 2003), which often display a stagnant status from 5 to 15 days after flowering (Zhang et al., 2019). Furthermore, Yang and Zhang (2010) demonstrated that the grain average weight and the average filling rate of inferior spikelets were 20.9% and 20.7% lower than those of superior spikelets, respectively. Besides, the poor activity of the involved enzymes in sucrose-to-starch metabolism (Mohapatra et al., 2009; Zhang et al., 2012) and lower hormone levels of ABA (Nonhebel and Griffin, 2020) are the major factors that contribute to poor grain filling. It has been previously documented that microRNA expression in seed development may cause poor grain filling of rice (Peng et al., 2014). Teng et al. (2022) demonstrated that starch synthesis and phytohormone biosynthesis were affected by differentially expressed microRNA leading to a decrease in rice yield. However, this standpoint currently lacks further support based on genetic evidence. In contrast, our research documented that the GF14f isoform of 14-3-3 proteins could be considered the hub of the regulatory network responsible for the poor grain filling in rice (Zhang et al., 2014; Zhang et al., 2015b; Zhang et al., 2017). Recently, the molecular mechanism has revealed that GF14f negatively affects rice grain filling in addition to interacting with key enzymes in sucrose-to-starch conversion (Zhang et al., 2019). Interestingly, the *GF14f* gene (encoding GF14f isoform of 14-3-3 proteins) was weakly expressed in the grains of ratoon rice during the time course of 7 to 28 days after flowering while taking a turn for highly up-regulated expression at 35 days, as compared to its counterparts in both early-season rice and late-season rice (**Supplementary Figure S1**). These results indicated the potential associations between differentially expressed *GF14f* and the grain filling process underlying rice ratooning.

Therefore, in this study, we analyzed the rice yield and grain quality comparing early-season rice, ratoon rice, and late-season rice with additional evaluation of carbon reserves remobilization. A further aim was to elucidate the key roles the *GF14f* gene has on carbon reserves remobilization, grain filling properties, starch composition, and structure. Finally, our aim was to understand whether gene-dependent regulation by *GF14f* had an impact on rice yield and quality improvement under rice ratooning, irrespective of seasonal or environmental effects.

## Materials and methods

### Experimental design and materials

*GF14f-RNAi line*, *GF14f mutant*, and their WT (Jinhui-809) were grown at the Experiment Station of Fujian Agriculture and Forestry University, Fuzhou, Fujian, China (119.280E, 26.080N). The *GF14f-RNAi line* was obtained by transgenic technology as previously described (Zhang et al., 2019), displaying a specific suppression of *GF14f* gene expression. The *GF14f* mutant was obtained from the T-DNA-inserted japonica line Dongjin, displaying the loss in expression of *the GF14f* gene (http://signal.salk.edu/cgi-bin/RiceGE). The suppression degree of the *GF14f-RNAi line* and *GF14f mutant* was investigated using qRT-PCR (**Figure S2**). Field trials were performed from March 2019 to October 2019 and repeated from March 2020 to October 2020. Weather data from 2019 and weather data from 2020 are provided in **Supplementary Figures S3**–**S4**. To provide evidence for revealing the key role of the *GF14f* gene in ratoon rice, the *GF14f-RNAi* line, *GF14f Mutant*, and the corresponding WT were specifically grown as ratoon rice and late-season rice, having the same or different genetic background and having a synchronized heading time. Planting and fertilization were conducted as in our previous research (Lin et al., 2022). These plant materials were provided by Fujian Provincial Key Laboratory of Agroecological Processing & Safety Monitoring, Fujian Agriculture and Forestry University, Fuzhou 350002, China.

### Evaluation of the ability of carbon reserve remobilization

The harvested rice plants were sampled at the heading stage and at maturity, respectively. Then, the plant materials were further divided into four tissue parts, which contained roots, stems, leaves, and grains. Here, the whole root, whole stem, whole leaf, and full-filled grains were respectively collected with their three replications to form a composite sample. Three composite samples were used as the three biological replications in the following experiments. Then, the samples were immediately dried in a forced-air dryer at 80 °C to constant weight for the estimation of dry matter weight (Ying et al., 1998). Stems and leaves were freeze-dried and ground to a fine powder for the estimation of non-structural carbohydrates (NSC). Total soluble sugar content was measured by a colorimetric method of anthracenone determination (Yang et al., 2001b; Wang et al., 2020). Total starch content was measured spectrophotometrically (Li and Peng, 2018). The absorbance was measured at 630 nm using a spectrophotometer (Model 340, Sequoia-Turner Co., Taiwan). Total NSC content was calculated as the sum of total soluble sugar and total starch. Other calculation formulas are as follows. Here, the transferable dry matter and transferable NSC were respectively referred to as the reduction of dry matter and the reduction of NSC in source tissues between the heading stage and the mature stage.


Dry matter translocation efficiency(%)=(1−(dry matters in source tissue at maturity/dry matters in source tissue at heading stage))×100%

Contribution of dry matter assimilation to grains(%)=(transferable dry matters/grain weight)×100%

NSC translocation efficiency(%)=(1−(NSC contents in source tissues at maturity/NSC contents in source tissues at heading stage))×100%

Contribution of NSC to grains(%)=(transferable NSC/grain weight)×100%

### Evaluation of yield performance and grain quality

At maturity, rice plants with uniform morphological features were harvested. Each panicle that had a seed setting percentage of over 50% was considered a productive panicle and grain weight was calculated based on 200 grains and was converted to 1,000-grain weight (Li et al., 2014). The seed-setting percentage, per-unit yield, and harvest index were calculated as previously described (Huang et al., 2020; Lin et al., 2022). Seed-setting percentage (%) = filled grains/(filled grains + partially filled grains + unfilled grains) × 100%, per-unit yield (t·ha^-1^) = total rice yield/whole-field area and harvest index (%) = grain dry weight/biological yield of overground parts × 100%. The panicles that were headed on the same day were tagged and then used to calculate the grain number per spike. The annual rice yield of ratoon rice was composed of the yield of early-season rice and the yield of ratoon rice. Daily average yield = per-unit yield/duration of the growing period.

Brown rice yield, milled rice yield, and head rice yield were counted according to the “Chinese agricultural standard: NY/T 83-2017” (Ministry of Agriculture of the PRC, 2017). Chalky grain percentage and chalkiness degree were measured using a grain scanner (ScanMaker i800 plus, Microtek, Shanghai) according to the manufacturer’s instructions. Chalky grain percentage = (numbers of chalky grains/numbers of total grains) × 100%. Chalkiness degree = (sum of chalky area/sum of whole grain area) × 100%.

### Assay of starch composition in the endosperm

Amylose content was measured using a continuous flow analytical system (Skalar San^++^ System, Netherlands). Standards were prepared using potato amylose (Solarbio, China) with a concentration gradient of 2%, 4%, 8%, 12%, 16%, 20%, and 32%. The absorbance of amylose content was determined at 600 nm. Total starch content was measured spectrophotometrically based on the determination of the absorbance of glucose concentration at 540 nm (Li and Peng, 2018). According to the transformational equation between starch and glucose, total starch content was calculated by the following formula: Total starch content (mg·g^-1^) = glucose concentration × 10 × 9.11 ml × 0.9/(50 × 1000). Amylopectin content = total starch content - amylose content (Prathap et al., 2019).

### Scanning electron microscopy analysis of the endosperm fine structure

According to the method described by Li et al., 2014, milled rice grains were cut in the middle and coated with gold under vacuum conditions. The starch morphology in the belly part of the endosperm was examined using a scanning electron microscope (Phenom ProX, FEI NanoPorts, America) at an accelerating voltage of 10 kV and a spot size of 30 nm.

### Transcriptomic and bioinformatics analysis

The *GF14f* mutant should be more genetically stable in the disturbing function of this gene than the *GF14f-RNAi line*. Therefore, we used the rice grains which were sampled from the *GF14f mutant* and its WT Dongjin for the following RNA sequencing. Total RNA was extracted from these two rice lines on the 7^th^ day after flowering. Grains from ten tagged panicles were collected and combined into an independent biological replication for each sample (at least 3 replicates). Total RNA was extracted using a TRIzol reagent (TransGen Biotech, China) and double-stranded cDNA was synthesized and purified using a QIAquick PCR extraction Kit (QIAGEN Inc., USA). The size-selected DNA of PCR products was ligated by pulse-field gel electrophoresis. We constructed cDNA libraries and completed Illumina sequencing (Illumina Hiseq™ 2500, America) at Majorbio company, China.

For further bioinformatic analysis, the reads with > 50% low-quality bases (Q-value of ≤30) or >10% of unknown nucleotides were removed (Lin et al., 2022). Transcriptome assembly was performed using the short-read assembly program Trinity (Grabherr et al., 2011). The unigene expression abundance was normalized to reads per kb per million reads (RPKM) using the following formula: RPKM = (1,000,000 × C)/(N × L/1000). Here, C is the number of reads that are uniquely mapped to one unigene, N is the total number of reads that are uniquely mapped to all unigenes and L is the length (base number) of one unigene (Fang et al., 2017). Further analysis and graphing were performed according to our previous research (Lin et al., 2021; Lin et al., 2022). More specifically, the edgeR package (http://www.r-project.org/) was used to identify differentially expressed genes (DEGs). Target proteins of DEGs were collectively annotated according to their numeric order in the GO database (http://www.geneontology.org/). GO terms with a p-value< 0.05 and an FDR< 0.05 were defined as the significant terms. GO enrichment analysis displayed the key DEGs that were clustered into their corresponding GO terms (https://www.omicshare.com).

### Assay of H_2_O_2_ concentration, ${\text{O}}_{2}^{-}$ production ratio, and MDA content

The crude proteins of grain samples were extracted at 4°C, and then H_2_O_2_ concentration, ${\text{O}}_{2}^{-}$ production rate, and MDA concentration were determined according to previously published methods (Ke et al., 2007; Chakrabarty and Datta, 2008).

### Data processing and statistical analysis

SPSS v. 25.0 (IBM Corp., Armonk, NY, USA) and Origin pro 2021 (OriginLab Corp., Northampton, MA, USA) were used for data processing and analysis of variance (ANOVA). Significant differences were evaluated with a p-value< 0.05.

## Results

### An extensive carbon reserve remobilization induced by rice ratooning

The ability to remobilize the carbon reserves significantly contributes to rice yield and grain quality. Here, both dry matter translocation efficiency and contribution of dry matter assimilation to grains in the different source tissues of ratoon rice were higher than in their counterparts in early-season rice and those in late-season rice. In 2019, the dry matter translocation efficiency in the root, stem, and leaf of the ratoon rice significantly (*P< 0.05*) increased by 7.9%, 24.4%, and 40.2%, respectively, as compared to early-season rice. Whereas, those parameters significantly (*P< 0.05*) increased by 9.0%, 37.5%, and 41.5% when compared to late-season rice. The contribution of dry matter assimilation to grains in the root, stem, and leaf of the ratoon rice significantly (*P< 0.05*) increased by 1.3%, 2.8%, and 2.4%, respectively, as compared to early-season rice. Furthermore, those parameters significantly (*P< 0.05*) increased by 1.1%, 2.9%, and 1.9% when compared to late-season rice. In 2020, a similar scenario was observed in the dry matter translocation efficiency and contribution of dry matter assimilation to grains, which were higher in the ratoon rice than those of early-season rice and of late-season rice. Consistently, both NSC translocation efficiency and contribution of NSC to grains in the different source tissues of the ratoon rice were also higher than in their counterparts in early-season rice and those in late-season rice. In 2019, the NSC translocation efficiency in the root, stem, and leaf of the ratoon rice significantly (*P< 0.05*) increased by 11.4%, 15.0%, and 16.5%, respectively, as compared to early-season rice. In contrast, those parameters significantly (*P< 0.05*) increased by 6.4%, 22.0%, and 13.0% when compared to late-season rice. In addition, the contribution of NSC to grains in the root, stem, and leaf of the ratoon rice significantly (*P< 0.05*) increased by 4.7%, 1.5%, and 2.0% when compared to early-season rice, while significantly (*P< 0.05*) increased by 4.1%, 1.7%, and 1.6% when compared to late-season rice. In 2020, the NSC translocation efficiency and the contribution of NSC to grains significantly (*P< 0.05*) increased in the root, stem, and leaf of the ratoon rice as compared to their counterparts in early-season rice and late-season rice (**Table 1**).

**Table 1:** Comparison of carbon reserve remobilization between early-season rice, ratoon rice, and late-season rice.

Years	Tissues	Cultivation system	Dry matter transference ratio (%)	Dry matter contribution ratio (%)	NSC translocation efficiency (%)	Contribution of NSC to grains (%)
2019	Root	MR	8.3 ± 0.2^b^	6.6 ± 0.2^b^	35.2 ± 2.7^c^	6.2 ± 0.7^c^
		RR	16.2 ± 0.3^a^	7.9 ± 0.2^a^	46.6 ± 2.4^a^	10.9 ± 1.0^a^
		LR	7.2 ± 0.3^c^	6.8 ± 0.3^b^	40.2 ± 5.7^b^	6.8 ± 0.7^b^
	Stem	MR	15.6 ± 0.1^b^	13.4 ± 0.2^b^	46.4 ± 3.8^b^	6.5 ± 1.1^b^
		RR	40.0 ± 0.2^a^	16.2 ± 0.2^a^	61.4 ± 1.4^a^	8.0 ± 0.4^a^
		LR	12.5 ± 0.1^c^	13.3 ± 0.2^b^	39.4 ± 4.3^c^	6.3 ± 1.3^b^
	Leaf	MR	16.5 ± 0.4^b^	10.4 ± 0.3^b^	39.9 ± 3.6^b^	2.9 ± 0.4^b^
		RR	56.7 ± 1.4^a^	12.8 ± 0.3^a^	56.4 ± 4.7^a^	4.9 ± 0.9^a^
		LR	15.2 ± 0.4^b^	10.9 ± 0.3^b^	43.4 ± 4.5^b^	3.3 ± 0.7^b^
2020	Root	MR	4.5±0.2^b^	3.4 ± 0.1^b^	36.2 ± 9.2^b^	4.2 ± 1.1^b^
		RR	9.2±0.7^a^	4.4 ± 0.3^a^	43.8 ± 5.8^a^	6.5 ± 0.8^a^
		LR	3.7±0.1^b^	3.4 ± 0.1^b^	33.3 ± 3.9^b^	4.2 ± 0.7^b^
	Stem	MR	16.0±0.2^b^	13.9 ± 0.2^b^	36.2 ± 2.5^b^	8.5 ± 0.6^c^
		RR	33.8±0.5^a^	16.3 ± 0.3^a^	73.0 ± 1.1^a^	11.2 ± 0.5^a^
		LR	12.7±0.3^c^	12.8 ± 0.4^c^	28.0 ± 2.8^c^	9.4 ± 0.4^b^
	Leaf	MR	12.4±0.3^b^	7.0 ± 0.2^b^	29.9 ± 4.0^b^	2.6 ± 0.5^b^
		RR	41.7±1.2^a^	8.9 ± 0.3^a^	42.0 ± 6.1^a^	3.2 ± 0.7^a^
		LR	10.5±0.3^b^	6.2 ± 0.1^c^	32.7 ± 6.3^b^	2.9 ± 0.7^b^

Superscript letters indicate statistical groups that are significantly different (P< 0.05, ANOVA) between early-season rice, ratoon rice, and late-season rice. Note: Jinhui-809 was used as the tested cultivar in the two-year experiment; MR, RR, and LR represent early-season rice, ratoon rice, and late-season rice, respectively.

These results suggested that rice ratooning could induce an extensive carbon reserve remobilization, thereby providing the grains with more carbon substances.

### Yield performance and grain quality improvement underlying rice ratooning

In our work, yield performance varied significantly between early-season rice, ratoon rice, and late-season rice. Between these treatments, panicle number, grain number per spike, 1000-grain weight, and per-unit yield all decreased in the ratoon rice. In contrast, the seed-setting percentage and harvest index of ratoon rice turned out to be much higher than those of early-season rice and of late-season rice. Furthermore, the results showed that the daily average yield of the ratoon rice was significantly (*P< 0.05*) higher than the daily average yield of early-season rice and of late-season rice. In 2019, the daily average yield of ratoon rice significantly (*P< 0.05*) increased by 28.1% and 28.6% when compared to early-season rice and late-season rice, respectively. And in 2020, the daily average yield of ratoon rice significantly (*P< 0.05*) increased by 16.6% and 22.6% when compared to main and late crops. It has also been found that the annual rice yield of ratoon rice (composed of main + ratooning crops) was significantly (*P< 0.05*) higher than the yield of late-season rice. In detail, the annual rice yield of ratoon rice significantly (*P< 0.05*) increased by 60.5% in 2019 and increased by 62.4% in 2020 (**Table 2**).

**Table 2:** Comparison of grain quality and yield performance between early-season rice, ratoon rice, and late-season rice.

Parameters	2019			2020		
	MR	RR	LR	MR	RR	LR
Brown rice yield (%)	86.9±0.4^a^	86.3±0.2^a^	87.0±0.2^a^	85.5±0.6^a^	85.5±0.8^a^	86.9±0.8^a^
Milled rice yield (%)	70.7±1.0^a^	72.2±0.9^a^	70.7±0.8^a^	73.9±1.0^a^	76.9±0.8^a^	74.1±1.0^a^
Head rice yield (%)	65.3±1.1^b^	67.4±1.4^a^	64.7±1.0^b^	66.3±0.8^b^	70.5±0.8^a^	65.7±1.3^b^
Chalky grain percentage (%)	6.2±0.3^a^	2.1±0.1^b^	5.3±0.4^a^	8.0±0.5^a^	2.7±0.1^b^	6.7±0.2^a^
Chalkiness degree (%)	5.3±0.9^a^	3.5±0.2^b^	5.0±0.3^a^	5.3±0.4^a^	2.2±0.2^b^	5.5±0.2^a^
Panicle number (Pcs)	13.2±1.7^a^	10.0±1.4^b^	12.8±1.2^a^	12.2±1.3^a^	10.6±1.1^a^	11.8±0.7^a^
Grain number per spike (Pcs)	275.6±5.1^a^	183.4±3.4^b^	273.6±10.8^a^	280.0±4.3^a^	162.6±3.3^b^	286.0±7.9^a^
1000-grain weight (g)	26.8±0.9^a^	24.7±0.9^b^	27.0±0.5^a^	27.2±0.4^a^	25.8±0.7^b^	27.2±0.3^a^
Seed-setting percentage (%)	81.8±1.8^b^	86.7±1.6^a^	82.2±1.1^b^	80.8±1.4^b^	85.3±0.7^a^	82.0±0.9^b^
Harvest index (%)	43.4±2.4^b^	51.3±2.0^a^	44.2±2.4^b^	41.8±1.6^b^	45.1±1.2^a^	41.6±1.3^b^
Per-unit yield (t·ha^-1^)	8.2±0.3^a^	5.6±0.1^b^	8.6±0.1^a^	8.5±0.3^a^	5.3±0.2^b^	8.5±0.4^a^
Daily average yield (kg·ha^-1^)	70.5±1.2^b^	90.3±1.8^a^	70.2±2.5^b^	73.1±1.5^b^	85.2±2.8^a^	69.5±0.5^b^
Annual rice yield (t·ha^-1^)	13.8 ± 0.4^a^		8.6±0.1^b^	13.8±0.5^a^		8.5±0.4^b^

Superscript letters indicate statistical groups that are significantly different (P< 0.05, ANOVA) between early-season rice, ratoon rice, and late-season rice, except for annual rice yield. The annual rice yield of ratoon rice was composed of two season yields and superscript letters here indicate statistical groups that are significantly different (P< 0.05, t-test) between main+ratooning crops and late-season rice. Note: Jinhui-809 was used as the tested cultivar in the two-year experiment; MR, RR, and LR represent early-season rice, ratoon rice, and late-season rice, respectively.

In addition, the key parameters of rice quality, such as brown rice yield, milled rice yield, head rice yield, chalky grain percentage, and chalkiness degree, were thoroughly investigated. In 2019, the head rice yield of the ratoon rice significantly (*P< 0.05*) increased by 2.1% and 2.3% when compared to early-season rice and late-season rice, respectively. However, the chalky grain percentage of the ratoon rice significantly (*P< 0.05*) reduced by 4.1% and 3.2% when compared to its counterparts of main and late crops. Likewise, the chalkiness degree of the ratoon rice significantly (*P< 0.05*) reduced by 1.8% and 1.5% when compared to early-cropping season and late-season rice, respectively. Consistently, in 2020, the head rice yield of the ratoon rice was significantly (*P< 0.05*) higher than the head rice yield of early-season rice and of late-season rice. In contrast, the chalky grain percentage and the chalkiness degree of ratoon rice were significantly (*P< 0.05*) lower than in their counterparts of early-season rice and late-season rice. Finally, the data did not display any statistical or biological differences in either brown rice yield or milled rice yield between early-season rice, ratoon rice, and late-season rice (**Table 2**).

In general, our results suggested distinct progress in improving head rice yield and grain chalkiness under rice ratooning. Moreover, the daily average yield of the ratoon rice was improved significantly by depending on the shorter growing period with the additional advantage of increasing the annual rice yield of ratoon rice.

### *GF14f* gene impacts on yield performance and grain chalkiness improvement underlying rice ratooning

**Figure 1:**
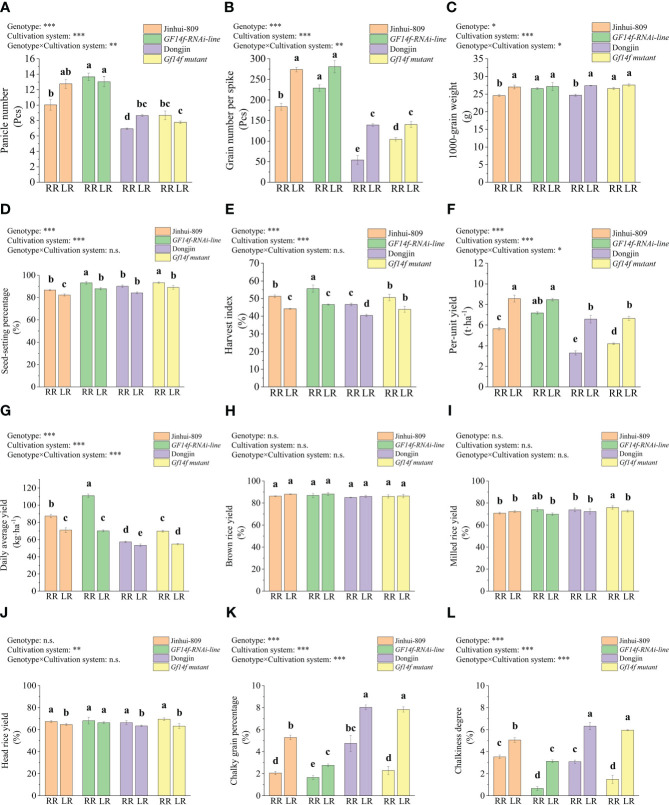
Comparisons in grain quality and yield performance between *GF14f-RNAi line*, *GF14f mutant* and their WT. **(A)**: Panicle number; **(B)**: Grain number per spike: **(C)**: 1000-grain weight; **(D)**: Seed-setting percentage: **(E)**: Harvest index; **(F)**: Per-unit yield; **(G)**: Daily average yield; **(H)**: Brown rice yield: **(I)**: Milled rice yield: **(J)**: Head rice yield: **(K)**: Chalky grain percentage: **(L)**: Chalkiness degree. Note: MR. RR and LR represent early-season rice, ratoon rice and late-season rice, respectively. Superscript letters indicate statistical groups that are significantly different (*P*< *0.05*, ANOVA). Asterisks indicate factors contributing to the differences between samples (n.s., not significant; *P< *0.05*; **P< *0.01*; ***P< *0.001*).

As was previously described above, per-unit yield in combination with panicle number, grain number per spike, and 1000-grain weight all performed at lower levels in the ratoon rice than their counterparts in late-season rice. However, these yield-related parameters strongly increased in either the *GF14f-RNAi line* or the *GF14f mutant*, respectively, as compared to their corresponding WT. In detail, panicle number, grain number per spike, 1000-grain weight as well as seed-setting percentage, harvest index daily average yield, and per-unit yield in ratoon rice of the *GF14f-RNAi* line significantly (*P< 0.05*) increased by 42.0%, 24.9%, 5.3% 6.3%, 4.3%, 7.3%, and 28.6% when compared to those in ratoon rice of the WT (Jinhui-809). Likewise, those yield-related parameters significantly (*P< 0.05*) increased by 25.0%, 92.5%, 7.6%, 3.1%, 4.0%, 21.7%, and 27.2% in ratoon rice of the *GF14f* mutant as compared to its WT (Dongjin) (**Figures 1A–G**).

In addition, the data did not display statistical and biological differences in brown rice yield, milled rice yield, and head rice yield between the *GF14f-RNAi line*, *GF14f mutant*, and their WT when grown as ratoon rice (**Figures 1H–J**). However, the low levels of chalky grain percentage and chalkiness degree in ratoon rice of the WT were significantly (*P< 0.05*) reduced by 0.4% and 2.8% in ratoon rice of the *GF14f-RNAi line*. Consistently, chalky grain percentage and chalkiness degree were significantly (P< 0.05) reduced by 2.5% and 1.6% in ratoon rice of the *GF14f mutant* as compared to those in ratoon rice of its WT. Chalky grain percentage and chalkiness degree in late-season rice of the WT also significantly improved (*P< 0.05*) when the *GF14f* gene function was suppressed (**Figures 1K–L**). Furthermore, ANOVA analysis indicated that most of the evaluative parameters (except brown rice yield, milled rice yield, and head rice yield) were simultaneously significantly influenced by two factors, namely cultivation system and genotype. The changes occurring in panicle number, grain number per spike, 1000-grain weight, per-unit yield, daily average yield, chalky grain percentage, and chalkiness were even affected by the cultivation system, genotype, and interaction effects between cultivation system and genotype (**Figure 1**).

Thus, these results suggested that yield performance and grain quality improvement underlying rice ratooning were strongly dependent on the *GF14f*, which negatively affected yield performance and grain chalkiness.

### *GF14f* gene impacts on carbon reserve remobilization and sucrose-to-starch biosynthesis underlying rice ratooning

**Figure 2:**
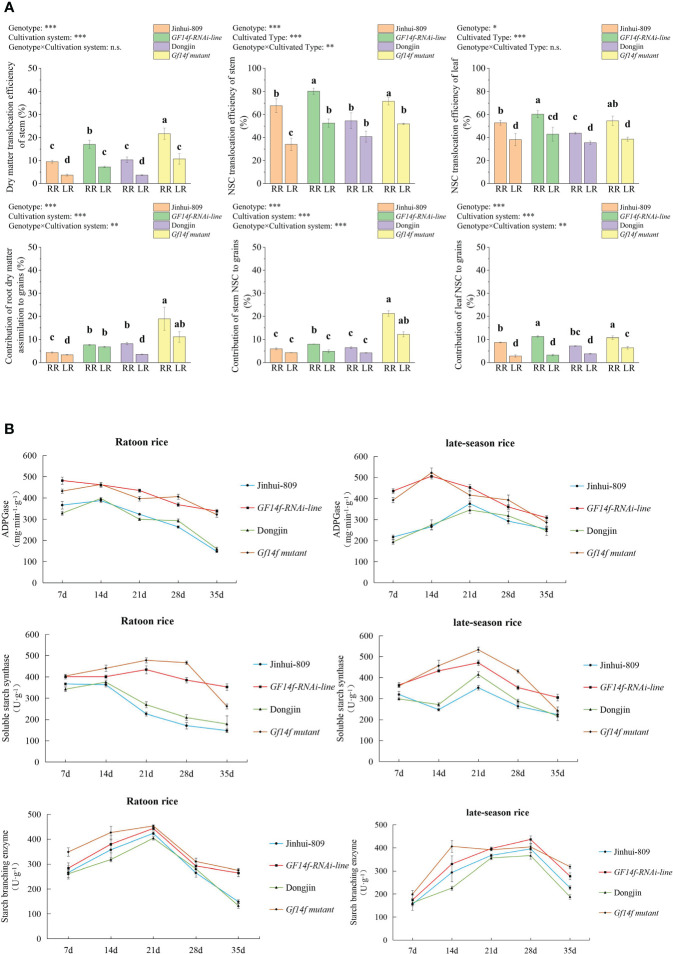
Comparisons in carbon reserve remobilization and sucrose-to-starch biosynthesis between *GF14f-RNAi line*, *GF14f mutant* and their WT. **(A)**: Carbon reserve remobilization in source tissues was mediated by the presence of the *GF14f gene*. Note: MR, RR and LR represent early-season rice, ratoon rice and late-season rice, respectively. Superscript letters indicate statistical groups that are significantly different (*P*< *0.05*, ANOVA). Asterisks indicate factors contributing to the differences between samples (n.s., not significant; *P< *0.05*; **P< *0.01*; ***P< *0.001*). **(B)**: Starch biosynthesis were enhanced by suppression of the *GF14f* gene. ADPGase, soluble starch enzyme and starch branching enzyme were essential for sucrose-to-starch biosynthesis.

As previously described, dry matter translocation efficiency and dry matter assimilation from root to grains were displayed at higher levels in the ratoon rice as compared to their low levels in late-season rice. A significant (*P< 0.05*) increase in dry matter translocation efficiency and dry matter assimilation to grains were further achieved in the root of the *GF14f-RNAi line* and the *GF14f mutant*, whether grown as ratoon rice or late-season rice, as compared to those in their counterparts of the WT. Moreover, NSC translocation efficiency and contribution of NSC from either stem or leaf to grains also displayed distinct improvements in the ratoon rice of the *GF14f-RNAi* line and the *GF14f* mutant, respectively (**Figure 2A**). During the grain-filling stage, the key enzymes in the sucrose-to-starch biosynthesis, which contained ADPGase, soluble starch synthase, and starch branching enzyme, reached greatly higher levels in not only ratoon rice but also in late-season rice when the *GF14f* gene was suppressed (**Figure 2B**).

**Figure 3:**
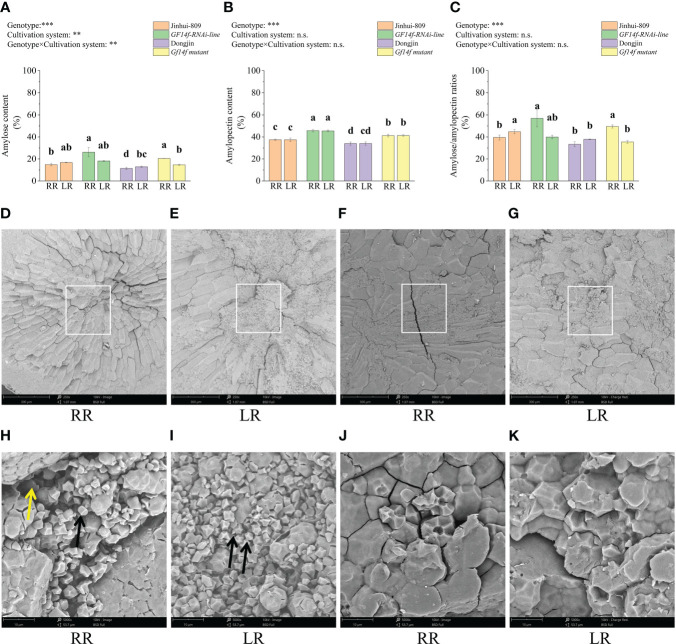
Comparisons in starch composition and scanning electron microscope observation in endosperm structure. **(A–C)**: Amylose, amylopectin and amylose/amylopectin ratio that varied between *GF14f-RNAi* line, *GF14f* mutant and their WT; **(D–K)**: Scanning electron microscopy images of the starch granules in endosperm. Positions marked by rectangles in the middle bellies of the endosperm. Scale bars: 250 times magnificention (top); 5000 times magnificention (bottom). Black arrows point loosened starch granules and yellow arrows point the loss of compositional parts of starch. MR, RR and LR represent early-season rice, ratoon rice and late-season rice, respectively.

Starch is the main substance for rice grain and scattered starch granules are the main contributing factor leading to poor grain quality. Here, amylose content, amylopectin content, and amylose/amylopectin ratio in the ratoon rice of the *GF14f-RNAi line* significantly (*P< 0.05*) increased by 11.3%, 8.3%, and 17.4%, respectively, when compared to those in the ratoon rice of the WT. Amylose content, amylopectin content, and amylose/amylopectin ratio in the ratoon rice of the *GF14f mutant* significantly (*P< 0.05*) increased by 9.0%, 8.1%, and 16.2% as compared to its WT (**Figures 3A–C**). Further analysis revealed that the ratoon rice of the *GF14f mutant* contained lower numbers and surface areas of scattered starch granules in the endosperm, displaying a complete and compact starch structure in contrast to the WT (**Figures 3D–K**).

Thus, these results indicated the regulatory roles of the *GF14f* gene negatively impacting carbon reserve remobilization and starch accumulation in the ratoon rice.

### *GF14f* gene modulates the gene-dependent plant resistance under rice ratooning

**Figure 4:**
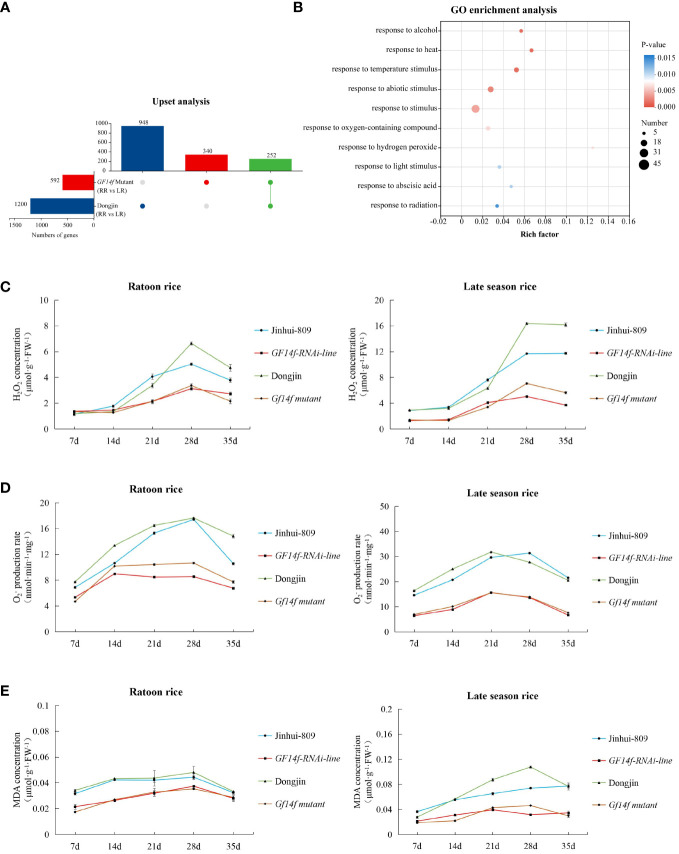
*GF14f gene* modulates the gene-dependent response to environmental stimuli underlying rice ratooning **(A)** Up-set analysis of *GF14 mutant* and its WT. **(B)**: Go enrichment analysis (TOP 10) of the 252 key DEGS which were differentially expressed in the pathways of both the *GF14f mutant* and the corresponding WT. GO database (http://www.geneontology.org/) was used to annotate the DEGs. The data showing the differentially expressed genes were visualized using Circos (Version 0.69, http://circos.ca/). Each gene was classified as belonging to their sample groups and was assigned to one special color in chordal diagram. **(C)** Temporal pattern of H_2_O_2_ concentration during grain-filling stage. **(D)** Temporal pattem of ${\text{O}}_{2}^{-}$ production rate during grain-filling stage. **(E)** Temporal pattern of MDA concentration during grain-filling stage.

We further investigated the regulatory roles for the *GF14f* encoded regulator underlying rice ratooning using transcriptome analysis. As a result, there were 948 differentially expressed genes (DEGs) significantly (|Fold change| > 2, *P< 0.05*) varying between ratoon rice and late-season rice in the WT (Dongjin). However, 340 DEGs that were significantly (|Fold change| > 2, *P< 0.05*) different could be detected when comparing ratoon rice and late-season rice using the *GF14F Mutant*. Among these, 252 DEGs were highly differentially expressed in both the *GF14F Mutant* and the WT (**Figure 4A**). Furthermore, the 252 DEGs were clustered into their corresponding GO terms (Top 10) using GO enrichment analysis. Among these, all the GO terms were involved in the processes of response to environmental stimuli and stress (**Figure 4B**). Thus, the differential expression of these functional genes could be considered as the potential cause leading to differentiation in stimuli-related plant resistance.

To further test the impact of the presence of the *GF14f* on plant resistance, the H_2_O_2_ concentration, ${\text{O}}_{2}^{-}$ production rate, and MDA concentration in rice grains were investigated. It could be shown that either the *GF14f-RANi line* or the *GF14f mutant* accumulated ROS and MDA during the grain-filling stage at very low levels. Interestingly, H_2_O_2_ concentration, ${\text{O}}_{2}^{-}$ production ratio, and MDA concentration in the late-season rice displayed lower levels when the *GF14f gene* was suppressed but showed much higher levels when monitoring their levels in late-season rice of the WT (**Figures 4C–E**). Furthermore, we performed qRT-PCR to investigate the differential gene expression of key genes regarding transcriptome analysis as described above. The results displayed that CAT-A, CAT-B, CAT-B (which encode catalase), OsAPX1, OsAPX2, OsAPX4 (which encode ascorbate peroxidase), and OsGPX1, OsGPX2, OsGPX4 (which encode glutathione peroxidase) greatly increased in the ratoon rice of the *GF14f mutant* when compared to those in the ratoon rice of the WT (**Figures S5A–I**).

Thus, these results indicated the essential roles of the *GF14f* gene in impacting plant resistance and ROS removal in the ratoon rice.

## Discussion

Ratoon rice cropping contributes to high rice yield and higher quality that can satisfy the present market demands (Xu et al., 2021), thereby increasingly becoming popular in worldwide rice production (Nakano et al., 2020; Wang et al., 2021; Xu et al., 2022). However, the potential mechanism affecting yield performance and grain quality underlying rice ratooning needs to be further investigated. In this study, a key gene, *GF14f*, impacting rice yield and quality as the regulatory hub in ratoon rice was explored for the first time. Furthermore, we discussed the essential roles and implications of how the *GF14f* gene impacts rice yield and grain quality under rice ratooning, irrespective of seasonal or environmental effects.

Carbon reserves remobilization is the basis for the grain filling of cereals leading to source-to-sink transport (Yang et al., 2006). Our finding demonstrated that rice ratooning was able to initiate extensive remobilization of carbon reserves to provide the grains with more carbon substances. Therefore, current knowledge (Yang et al., 2001a; Yang et al., 2001b; Wang et al., 2020) allows us to infer the following scenario—yield performance and grain quality in the ratoon rice are predicted to achieve higher levels. In our work, chalky grain percentage and chalkiness degrees were highly improved when rice plants were grown as ratoon rice. This result indicated a distinct improvement in rice appearance quality under rice ratooning. However, per-unit yield in combination with panicle number, grain number per spike, and 1000-grain weight in the ratoon rice were significantly reduced when compared to those in the early-season rice and those in the late-season rice. It seems that rice ratooning in particular contributes to obtaining higher grain quality rather than higher yield. However, this is only true if applied universally without considering the growing period. Under normal circumstances, the duration of the growing phase of traditionally-cultivated rice was longer than the duration of the growing phase of ratoon rice (Wang et al., 2020; Xu et al., 2021). Rice ratooning initiated grain filling once the new spikes were regenerated from auxiliary buds on the residual stubbles (Lin et al., 2015; Lin, 2019), thereby reducing the duration of the vegetative growth period. We, therefore, determined the daily average yield of ratoon rice. Consequently, the daily average yield was highly improved when monitoring ratoon rice. In addition, seed-setting percentage and harvest index were both displayed at higher levels in the grains of ratoon rice than their levels in either early-season rice or late-season rice. Not only that, the annual rice yield of ratoon rice (composed of main + ratooning crops) was much higher than the yield of single-cropping late rice. Thus, ratoon rice cropping is especially applicable for greater rice production in Southern China because light and temperature resources are enough for one seasonal crop but insufficient for two seasons (Lin et al., 2015; Huang et al., 2020; Xu et al., 2022). Southern China, which makes the greatest contribution to China’s rice industry, has currently been constrained by labor shortage (Deng et al., 2019), water scarcity (Zhuang et al., 2021), negative environmental impacts (Zhang et al., 2015a; Shen et al., 2021), and a lower economic return (Yu et al., 2021). Therefore, our finding provides compelling evidence to highlight the significance of ratoon rice cropping in Southern China. Importantly, such reductions involving per-unit yield, panicle number, grain number per spike, and 1000-grain weight of ratoon rice were highly improved when the *GF14f* gene was thoroughly suppressed. Additionally, the original high-quality of ratoon rice was further improved when investigating the *GF14f-RNAi line* and the *GF14f mutant*. A further significance was to observe how it was possible to achieve higher levels of yield performance and grain quality of ratoon rice via suppression of the *GF14f* gene. Furthermore, all analyses were conducted based on the comparison between ratoon rice and late-season rice, having the same or a different genetic background and the synchronized rice heading time so as to remove the seasonal and environmental effects. Therefore, our finding provided the basis for a hypothesis that the gene-dependent effect by the *GF14f* determines the yield performance and grain quality underlying rice ratooning.

Grain filling of cereals is an essential process catalyzed by a series of enzymes from sucrose-to-starch biosynthesis (Yang et al., 2003; Yang et al., 2004; Tang et al., 2009). Carbon substances were quickly transferred from source tissues into grains (Yang et al., 2001a; Yang et al., 2001b). In our work, the carbon reserves remobilization in the ratoon rice of the *GF14f-RNAi line* and the *GF14f mutant* were also further improved when compared to their WT. Meanwhile, the key enzymes in sucrose-to-starch biosynthesis were kept at high levels during the whole grain-filling stage when the *GF14f gene* was suppressed. It should be noted that high yield and rice quality are positive results ensuing from a continued high enzymatic activity of the key enzymes in starch biosynthesis (Yang et al., 2003; Yang et al., 2004; Yang et al., 2006). In addition, it was previously documented that starch is the most abundant and essential element in rice grain and that it greatly affects grain quality (Burrell, 2003; Bao, 2019), and this trait was influenced by starch composition, structure, and properties (Gu et al., 2019; Peng et al., 2021; Zhu et al., 2021). Furthermore, the currently available data in our work suggested that starch compositions varied significantly between the *GF14f-RNAi line*, the *GF14f mutant*, and their corresponding WT and, ultimately, displayed lower numbers and surface areas of scattered starch granules in the endosperm of both the *GF14f-RNAi line* and the *GF14f mutant*. It has been documented that lower numbers and surface areas of scattered starch granules in combination with a lower amylose/amylopectin ratio significantly contributed to reduced grain chalkiness (Wani et al., 2012; Schirmer et al., 2013; Li et al., 2014; Lin et al., 2018). This is consistent with our findings, thereby indicating a negative regulatory role of the *GF14f* gene, which impacted starch composition and structure in the endosperm. Our findings, therefore, indicated that the *GF14f* negatively affected several essential processes in ratoon rice, namely carbon reserve remobilization, grain filling process, and starch biosynthesis. Nonetheless, the in-depth molecular mechanism associated with these processes needs to be further investigated. In this study, a transcriptome analysis of the *GF14f mutant* and its WT would provide a more comprehensive understanding of how the *GF14f* regulates the grain filling of ratoon rice at the transcriptional level. Consequently, the data was suggested to be involved in the process of stimuli-related plant resistance. Environmental stimuli are the main cause of poor grain filling (Lawas et al., 2018; Yao et al., 2020), and oxidative and environmental stress were easily induced by many environmental stimuli, which led to excessive accumulation of ROS and MDA (Kaneko et al., 2016; Sharma and Sharma, 2018). In addition, carbon reserve remobilization and grain filling are long-term processes with accompanying cell metabolism, thereby producing several toxic compounds such as ROS. These were especially true when examining the loss of rice yield and grain quality (Xing and Zhang, 2010; Hakata et al., 2012; Lo et al., 2016; Suriyasak et al., 2017). However, both the *GF14f-RNAi line* and the *GF14f mutant* were better at increasing oxidative and environmental stress resistance as compared to their WT *via* reducing ROS accumulation and gene expression of the involved gene. Our findings, therefore, indicated that the *GF14f* gene can modulate the gene-dependent plant resistance and ROS removal in grains underlying rice ratooning. In addition, we also investigated the key genes that were documented to be essential for the chalky grain process and yield-related process. As a result, these key genes were also highly expressed in the *GF14f mutant* using a qRT-PCR analysis.

In summary, this study revealed the yield performance and grain chalkiness improvement underlying rice ratooning. Such processes were further shown to be associated with a key gene, *GF14f.* The *GF14f* had a negative impact on many aspects ranging from carbon reserve remobilization, starch biosynthesis, stimuli-related plant resistance, and ROS removal. The yield performance and grain quality were, therefore, dependent on the *GF14f* gene, irrespective of seasonal or environmental effects.

## Data availability statement

The original contributions presented in the study are included in the article/**Supplementary Material**. Further inquiries can be directed to the corresponding author.

## Author contributions

FL and WL contributed to the conception of the study; FL performed the experiment; SL and ZZ contributed to the manuscript preparation; FL performed the data analyses and wrote the manuscript; CR and DX helped to work out the conception and analysis with constructive discussions. They also helped revise the initial draft. All authors contributed to the article and approved the submitted version
