# Supplementary material for: GF14f gene is negatively associated with yield and grain chalkiness under rice ratooning
Source: Front Plant Sci. 2023 Feb 10;14:1112146. doi: 10.3389/fpls.2023.1112146 (PMC9976807; doi:10.3389/fpls.2023.1112146)
Supplement: Supplementary file 1 [file DataSheet_1.pdf]

GF14f gene is negatively associated with yield and grain chalkiness under rice ratooning

Feifan Lin[1], Sheng Lin[2,3], Zhixing Zhang[2,3], Wenxiong Lin[2,3,*], Christopher Rensing[4,*] & Daoxin Xie[1,*]

[1] Tsinghua-Peking Joint Center for Life Sciences, and MOE Key Laboratory of Bioinformatics, School of Life Sciences, Tsinghua University, Beijing,100084, China

[2] Fujian Provincial Key Laboratory of Agroecological Processing and Safety Monitoring, School of Life Sciences, Fujian Agriculture and Forestry University, Fuzhou 350002, China

[3] Key Laboratory of Crop Physiology and Molecular Ecology, Fujian Agricultural and Forestry University, Fuzhou 350002, China

[4] Institute of Environmental Microbiology, College of Resources and Environment, Fujian Agricultural and Forestry University, Fuzhou 350002, China

*Corresponding authors:Daoxin Xie; e-mail: daoxinlab@mail.tsinghua.edu.cn, Christopher Rensing; e-mail: rensing@iue.ac.cn, Wenxiong Lin; e-mail: lwx@fafu.edu.cn.

Supplementary Information:
Including 5 supplementary figures.

Figure S1

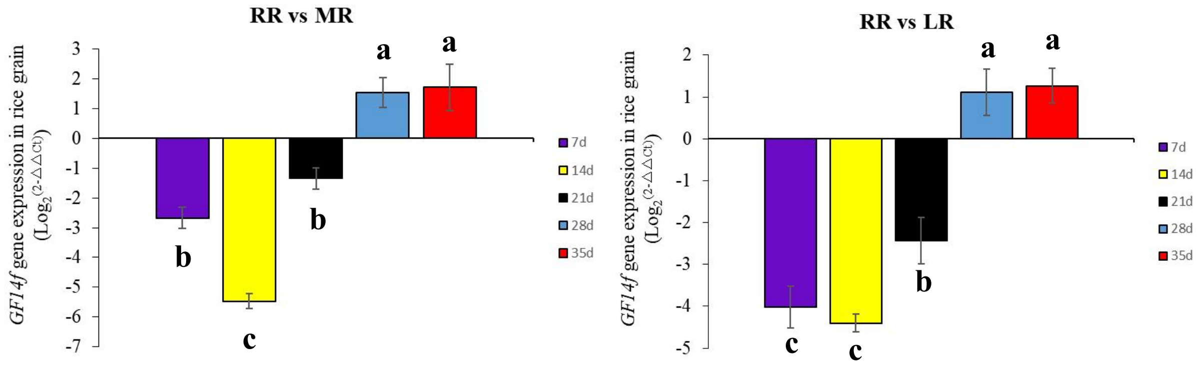

Fig. S1. The relative expressions of *GF14f* in rice grain. (A): Gene expression of *GF14f* in ratoon season rice that was compared to early-season rice. (B): Gene expression of *GF14f* in ratoon rice that was compared to late-season rice. Gene expression was calculated using $2^{\wedge-\Delta\Delta Ct}$ method and normalized with respect to the Ct value of *β-Actin,* estimated by $Log_2^{(2^{\wedge}-\Delta\Delta Ct)}$. Note: RR, MR and LR represents ratoon rice, early-season rice and late-season rice, respectively. Here, early-season rice represents the first season rice in ratoon cropping system and late-season rice represent was specially grown as the same genetic background and having a synchronized heading time as ratoon rice. Superscript letters indicate statistical groups that are significantly different ($P < 0.05$, ANOVA).

Figure S2

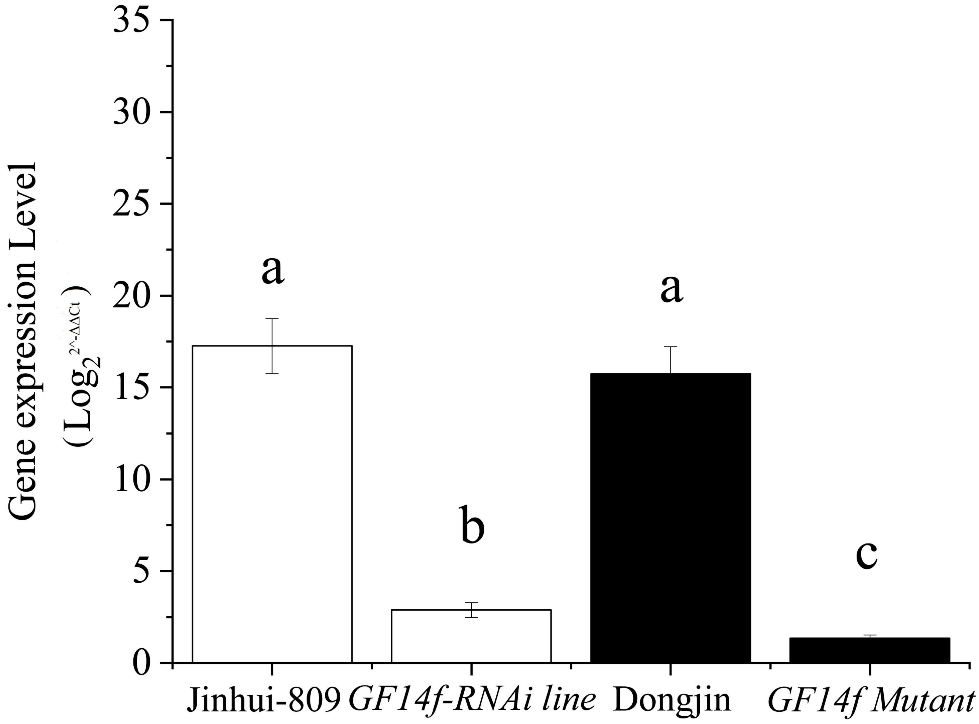

Fig. S2. The suppression degree of GF14f gene in *GF14f-RNAi line* and *GF14f mutant*. Note: Gene expression was calculated using $2^{\wedge-\Delta\Delta Ct}$ method and normalized with respect to the Ct value of β-Actin, estimated by $Log_2^{(2^{\wedge-\Delta\Delta Ct})}$. Superscript letters indicate statistical groups that are significantly different ($P < 0.05$, ANOVA).

Figure S3

A

B

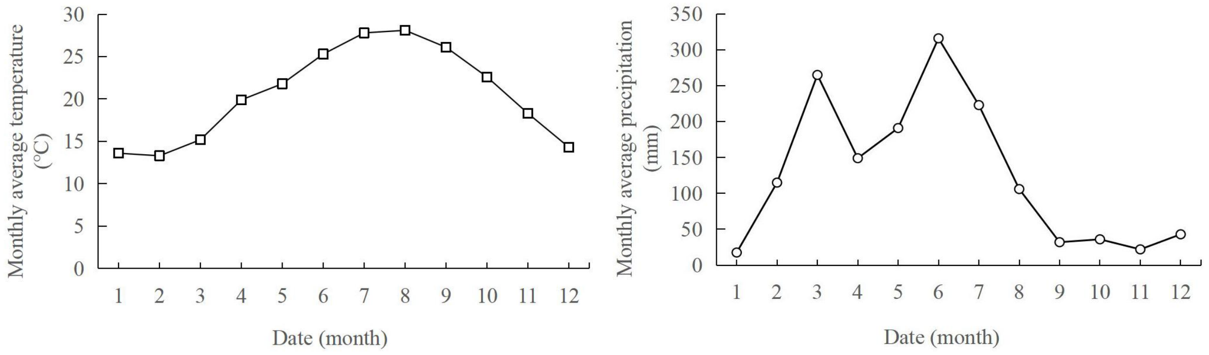

Fig. S3. Monthly Weather Data 2019. (A) Monthly average temperature. (B) Monthly average precipitation.

Figure S4

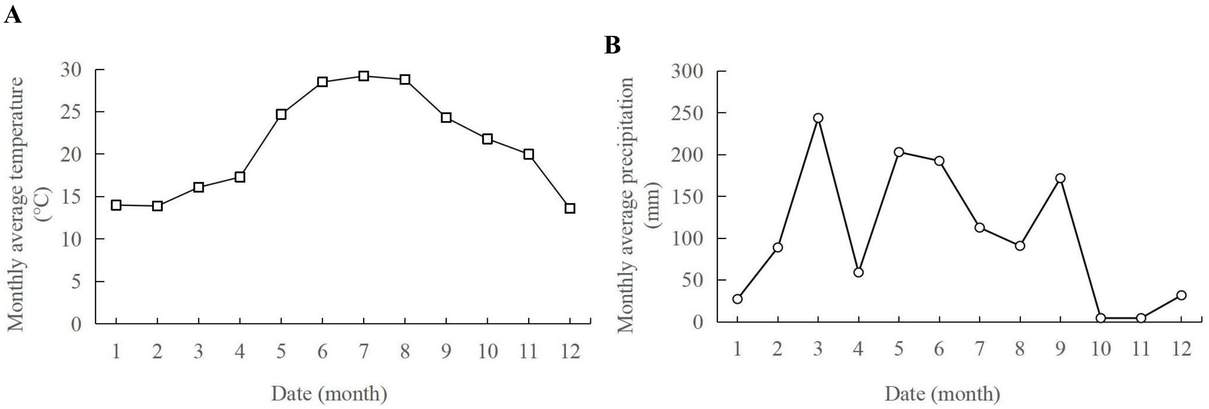

Fig. S4. Monthly Weather Data 2020. (A) Monthly average temperature. (B) Monthly average precipitation.

Figure S5

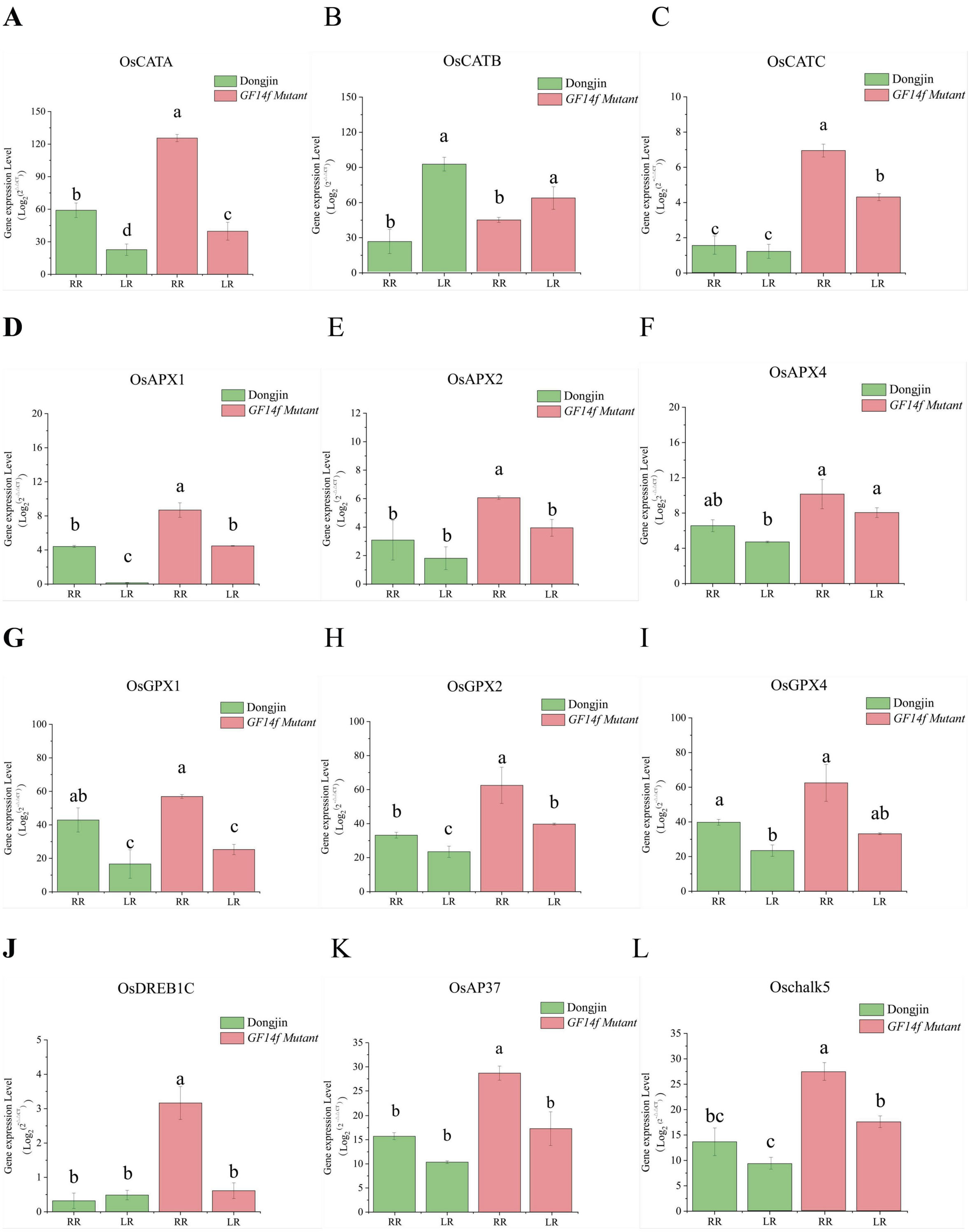

Fig. S5. The gene expression of those key genes related to ROS removal and plant resistance, rice yield and grain quality. Note: Gene expression was calculated using $2^{\wedge -\Delta\Delta Ct}$ method and normalized with respect to the Ct value ofβ-Actin, estimated by $Log_2^{(2^{\wedge -\Delta\Delta Ct})}$. Superscript letters indicate statistical groups that are significantly different (*P <0.05*, ANOVA).
